# Acid Red 66 Dye Removal from Aqueous Solution by Fe/C-based Composites: Adsorption, Kinetics and Thermodynamic Studies

**DOI:** 10.3390/ma13051107

**Published:** 2020-03-02

**Authors:** Camila B. Paz, Rinaldo S. Araújo, Lais F. Oton, Alcineia C. Oliveira, João M. Soares, Susana N. Medeiros, Enrique Rodríguez-Castellón, Elena Rodríguez-Aguado

**Affiliations:** 1 Instituto Federal de Educação, Ciência e Tecnologia do Ceará, IFCE Campus de Fortaleza, Av. 13 de Maio, 2081-Benfica, CEP 60040-531 Fortaleza, Ceará, Brazil; milabpaz@gmail.com; 2 Departamento de Química Analítica e Físico-Química, Campus do Pici-Bloco 940, Universidade Federal do Ceará, 60040-531 Fortaleza, Ceará, Brazil; laisoton9@gmail.com; 3 Departmento de Física, Universidade do Estado do Rio Grande do Norte-UERN, BR 110-km 48, R. Prof. Antônio Campos, Costa e Silva, 59610-210 Mossoró, Rio Grande do Norte, Brazil; joaomsoares@gmail.com; 4 Departamento de Física, Universidade Federal do Rio Grande do Norte, Av. Senador Salgado Filho, 3000, 59075-000 Natal, Rio Grande do Norte, Brazil; 5 Departamento de Química Inorgánica, Facultad de Ciencias, Universidad de Málaga, 29071 Málaga, Spain

**Keywords:** acid red 66 dye, composites, Freundlich, Mössbauer, Langmuir, magnetic particles

## Abstract

The presence of synthetic dyes in water causes serious environmental issues owing to the low water quality, toxicity to environment and human carcinogenic effects. Adsorption has emerged as simple and environmental benign processes for wastewater treatment. This work reports the use of porous Fe-based composites as adsorbents for Acid Red 66 dye removal in an aqueous solution. The porous FeC and Fe/FeC solids were prepared by hydrothermal methods using iron sulfates and sucrose as precursors. The physicochemical properties of the solids were evaluated through X-ray diffraction (XRD), Scanning electron microscopy coupled with Energy dispersive spectroscopy (SEM-EDS), X-ray photoelectron spectroscopy (XPS), Fourier transform infrared s (FTIR), Raman and Mössbauer spectroscopies, nitrogen adsorption–desorption isotherms, Electron Paramagnetic Resonance (EPR) and magnetic saturation techniques. Results indicated that the Fe species holds magnetic properties and formed well dispersed Fe_3_O_4_ nanoparticles on a carbon layer in FeC nanocomposite. Adding iron to the previous solid resulted in the formation of γ-Fe_2_O_3_ coating on the FeC type structure as in Fe/FeC composite. The highest dye adsorption capacity was 15.5 mg·g^−1^ for FeC nanocomposite at 25 °C with the isotherms fitting well with the Langmuir model. The removal efficiency of 98.4% was obtained with a pristine Fe sample under similar experimental conditions.

## 1. Introduction

The synthetic dyes are widely used by several industries such as textile, painting, leather and printing. The discharge of the dyes in water effluents continues to be one of the greatest health problems worldwide, affecting human health and the environment [1,2,3]. Of particular importance, the textile dyes are typically composed by organic molecules from colored fabrics, which uses large amounts of these organic compounds for textile production to fulfill the consumer needs [2,3]. However, the organic dyes can cause several damage and environmental issues, when they are not appropriately handle [3,4]. Noteworthy, the absorption of the sunlight by wastewater from textile dyes hinders light penetration into water and thus, reducing the possibility of photosynthesis; as a consequence, a lack of dissolved oxygen to sustain aquatic life is observed [2,5].

Additionally, most of the synthetic dyes are non-biodegradable causing harm to human health, when expose directly to this type of dye wastewater [3,5]. These problems accelerate rules and strict regulations for textile dyeing industries promoting a scientific interest to achieve environmental sustainable chemical routes and protocols to removal of dye colorants in textile wastewater [6,7]. 

In recent years, several methods for removal of synthetic dyes removal, including ion exchange, coagulation−flocculation, electrochemical processes, membrane technology, irradiation, biological treatment with microbes, chemical oxidation and adsorption have been studied [8,9,10]. The adsorption process is proved to be a promising strategy to attack this remediation problem in synthetic dye removal from wastewater, when considering its broad spectrum and ability to work in scaling conditions [3,11,12]. 

Many materials have been used as adsorbents including oxides, hydrogels, activated carbon, zeolites, graphene oxide, composites, polymers and carbon nanotubes [13,14,15]. Among the various adsorbents, the iron-based oxides seems to be most studied due to its reusability, nontoxicity, elevated adsorption capacity, long-term stability, low cost and facile removal of the adsorbent from the solutions without generate secondary pollution by leaving other chemical components in wastewater as by-product [2,12,15]. 

The iron particles are regarded as very efficient adsorbents for wastewater treatment due to their intrinsic magnetism [16]. This enables the application of an external magnetic field to remove pollutants from wastewater solutions. Besides, the presence of π=π aromatic bonds, –SO_3_^–^ and –NH_2_ groups in an anionic organic dye might form weak interactions with iron particles on its surface [16]. 

Additionally, iron-oxide nanoparticles provided of magnetic behavior, e.g., magnetite and maghemite could be assembled to inorganic supports following diverse approaches giving rise to inorganic-inorganic composites. An effective procedure is based on the impregnation of porous solids, i.e., silica, clays, zeolite, activated carbon, among others, using a non-aqueous dispersion of oleic acid-modified magnetite particles. For instance, the use of magnetite in the form of a ferrofluid allows a homogenous distribution on the solid’s surfaces, maintaining their adsorbent properties together with superparamagnetic behavior at room temperature and therefore, facilitating the elimination of pollutants in water by means of an external magnetic field. Typically, the powerful adsorbent sepiolite fibrous clay, modified with magnetite, can retain organic dyes and further removed by using a magnet [17]. Similar multifunctional nanostructured platforms containing magnetite particles and Prusian blue are highly effective adsorbents for the removal of cesium ions from aqueous solution, even in the presence of sodium ions with the advantage of allowing an easy recovery of the cesium-loaded materials with the help of a magnet [18]. 

Therefore, the surface charges should be modulated to produce a stronger electrostatic attraction to efficiently remove the anionic dye over iron-oxides particles. This type of dye adsorption with using on iron species is usually achieved by fixing the Fe particles onto a carbon material that gives a fast separation of the surface charge and its further regulation of the pollution [13,16]. Although, a complete chemical mechanism of dye removal and its adsorption rates improvement in the iron species have not been establish yet, the potential route for wastewater with dye organic molecules treatment in wastewater is needed. 

Due to strong motivation for the use environmentally friendly iron-based materials adsorbents for wastewater treatment, this work aims to synthesize Fe-based composites for Acid Red 66 dye removal from aqueous solutions. The Ponceau BS dye so called Acid Red 66, is an acid anionic azo compound, which is used widely in the dying processing industry [19,20]. In addition, the Acid Red 66 dye chemical structure contains nitrogen to nitrogen, i.e., –N=N– bonds with the aromatic rings mostly substituted by sulfonate groups. Therefore, the Acid Red 66 is very suitable to be absorbed by the Fe-based adsorbents possessing positive surface charge in the acidic solution media [16,19,20]. 

To date, scientific reports to use the proposed iron-based adsorbents for decolorization of Acid Red 66 are scarce. This study presents a detailed investigation about preparation of the composites, morphological and crystallographic characterizations, some of adsorption kinetics and thermodynamics with sole intention to prove a potential application of the Fe-based composites. Particularly, the advantages of the Fe-based composites prepared in this study consist of their low-cost, high adsorption capacity and easy regeneration only using a magnet and thereby, avoiding further separation of the adsorbent steps. Therefore, the solids in study are environmentally friendly adsorbents and alternatives for the remediation problem in wastewater. 

## 2. Experimental 

### 2.1. Materials

Ferrous sulfate heptahydrate (FeSO_4_·7H_2_O), ferric sulfate anhydrous (Fe_2_(SO_4_)_3_, 99%), anhydrous sucrose (C_12_H_22_O_11_, 99%) and aqueous ammonia (NH_3_(aq), 28%) were purchased from Vetec (Vetec, São Paulo, Brazil). The Acid Red 66 dye was purchased from Sigma-Aldrich (Sigma-Aldrich, St. Louis, MO, USA), All reactants were used as received.

### 2.2. Preparation of the Fe-Based Composites 

**Scheme 1:**
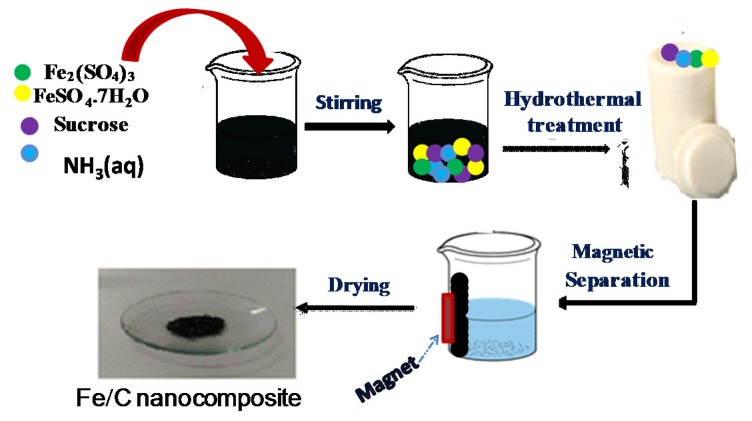
Illustration of the diagram depicting the preparation of the composites.

Ferrous sulfate heptahydrate (FeSO_4_·7H_2_O) and ferric sulfate anhydrous (Fe_2_(SO_4_)_3_, 99%, Vetec), anhydrous sucrose (99% Vetec) and aqueous ammonia (NH_3_(aq), 28% Vetec) were used as starting materials for the preparation of the composites. In a typical synthesis, 3.4 g Fe_2_(SO_4_)_3_, 1.2 g of FeSO_4_·7H_2_O and 7.0 g of sucrose were precipitated with 9.0 mL of aqueous ammonia, based on the methodology described by Liu et al. [21], with modifications. Afterwards, the mixture possessing a pH of 12 was placed into an autoclave with a volume of 40 mL. Subsequently, the autoclave was kept at 150 °C in a constant temperature oven for 1 day and then, cooled naturally to room temperature. Then, a black precipitate was formed and removed by using a magnet, being afterwards washed thoroughly with distilled water and separated by magnetization procedure. After that the as-synthesized product placed to dialysis with a membrane for 6 days to remove the excess of sucrose and sulfates ions. The final pH obtained was 6.8. Finally, the obtained product was dried at 90 °C for 2 h with formed black powder having Fe^3+^ to Fe^2+^ molar ratio of 4. The solid was designed as FeC composite, according to the Scheme 1. 

To prepare the Fe/FeC composite, about 100 mL of a 1 mol·L^−1^ Fe(NO_3_)_3_·9H_2_O solution was added to 1 g of the previous FeC solid, under stirring. The mixture remained during 1h under aging. After this period, the obtained powder was thoroughly washed with ultrapure water to attain the electrical conductivity of zero value, followed with drying process at 90 °C for 2 h. 

A reference Fe sample was synthesized without sucrose in the presence of Fe_2_(SO_4_)_3_, FeSO_4_·7H_2_O and aqueous ammonia precursors. After submitting the mixture to hydrothermal treatment under the same conditions above mentioned, the Fe sample was obtained.

### 2.3. Characterizations 

Powder X-ray diffraction (XRD) patterns were estimated using a DBMAXB Rigaku diffractometer (Bruker, Karlsruhe, Germany) with Cu_K__α_ radiation at 40 kV and 40 mA. The intensity data was collected over a 2θ value from 20° to 70°. The patterns were indexed using Joint Committee of Powder Diffraction Standard (JCPDS) data base files. 

Vibrational modes were obtained using Raman spectroscopy with a LabRam spectrometer (HR Horiba Scientific), which is equipped with a charge coupled device. The detector was cooled using liquid nitrogen at −196 °C. An excitation source line of 532 nm was used with the laser power of 20 mW. The spectral resolution was 4 cm^−1^ with the use of an objective lens of 100 times. The spectra were recorded in the 100–200 cm^−1^ range. 

Fourier transform infrared spectroscopy (FTIR) measurements were carried out in the 500–4000 cm^−1^ range in the Spectrum 100 Perkin Elmer equipment (Bruker, Rheinstetten, Germany). About 10 mg of the samples were mixed with 200 mg of vacuum dried KBr and subjected to external pressure of 8 tons on each sample, in order to create an almost transparent pill to perform FTIR measurements. 

The morphology was observed using a FEI, Quanta 200 FEG electron microscope (FEI Quanta, Hillsboro, OR, USA) in scanning electron microscopy (SEM) mode. The unit is equipped with Energy Dispersive Spectroscopy (EDS) system and all samples were gold sputtered to achieve a good contrast. 

Textural properties of the solids were evaluated through nitrogen adsorption–desorption isotherms at liquid nitrogen temperature in an ASAP 2420 apparatus from Micromeritics (Micrometrics, Norcross, GA, USA). Previously, the samples were heated at 150 °C and outgassed at this temperature for 8 h. Specific surface areas were calculated by using the Brunauer Emmett and Teller (BET) equation and the pore size distribution were obtained by the Barrett Joyner Halenda (BJH) model.

The chemical states of the elements were determined by X-ray photoelectron spectroscopy (XPS). The XPS spectra were recorded with a Physical Electronics VersaProbe II Scanning XPS Microprobe (Minneapolis, MN, USA) with scanning monochromatic X-ray Al Kα radiation (100 m area analyzed, 52.8 W, 15kV, 1486.6 eV), and a charge neutralizer. The pressure in analysis chamber was maintained lower than 2.0 × 10^–6^ Pa. High-resolution spectra were recorded at a given take-off angle of 45° by a multi-channel hemispherical electron analyser operating in the constant pass energy mode at 29.35 eV. The energy scale was calibrated using Cu 2*p*_3/2_, Ag 3*d*_5/2_, and Au 4*f*_7/2_ photoelectron lines at 932.7, 368.2 and 83.95 eV, respectively. Spectra were charge referenced with the C 1*s* of adventitious carbon at 284.8 eV.

Zeta potential analyses were carried out in a Zetasizer Nano ZS instrument (Malvern, GBR, Cambridge, UK) equipment. About 0.03 g of the samples in 30 mL of ultrapure water was used. The solids were placed in vials and the pH was 6.8. Samples were taken from the supernatants to perform the zeta potential analyses. 

Electron paramagnetic resonance (EPR) measurements were carried out to detect the local environment of iron atoms and valence states. The EPR measurements were performed using a Bruker spectrometer (Bruker, Rheinstetten, Germany) with X-band microwave frequencies at 9.5 GHz with sweep integrated for X-band spectra to determine the *g* factor. Before the measurements, all samples were flushed with helium at room temperature. 

Magnetic properties of the composites were evaluated through the magnetic hysteresis loops using Lakeshore vibrating sample magnetometer (VSM). The analyses were conducted in a Mini 5T Cryogenic equipment. Previously, the VSM was calibrated using YIG spheres to obtain the magnetization curves at room temperature.

Structural and magnetic features of the solids were investigated by ^57^Fe Mössbauer spectroscopy. The measurements were performed at room temperature with a Wissel spectrometer working in constant acceleration mode. The measurements were carried out at room temperature and using a ^57^Co/Rh source in transmission geometry with a radioactive source and activity of 50 mCi. The spectra were fitted using the Fit routine by a Lorentzian profile peaks. The isomer shift (δ), electric quadrupole splitting (Δ), hyperfine magnetic fields (BFH) and relative abundance were acquired from the Mössbauer spectra. The calibration of velocity scales was performed with α-Fe and all isomer shifts are given with respect to the Mössbauer spectra of the aforesaid iron phase. 

### 2.4. Adsorption Studies

Batch adsorption experiments were performed to test the potentiality of the composites on degradation of Acid Red 66 dye. All dyes adsorption studies were carried out in 50 mL dye-containing solution with a concentration of 2 g·L^−1^ at 25 °C. The 100 mg of iron composites were used at pH to 6.8 under vigorous stirring conditions of 150 rpm for 2 h to ensure the extensive adsorption equilibrium. The mixture was separated by magnetization and collected using filtered liquor to measure dye concentration by UV-Vis technique with an Evolution 60S^®^ Thermo Scientific equipment (Waltham, MA, USA). For comparison, each adsorption experiment was conducted twice to depict any adsorption behavior of Acid Red 66 dye by iron-based composites.

The adsorption experiments were performed to evaluate the thermodynamic effects and to obtain adsorption isotherms. Also, studied on the amounts of adsorbed dye (mg·g^−1^) were determined from their concentrations, before and after adsorption, by applying Equation (1):

(1) $$q=\frac{(C_{eq}-C_{o})\times V}{m}$$

where *C_o_* and C*_eq_* are the initial and final values of the dye concentration (mg·L^−1^), respectively. The volume (L) of the dye solution is represented by *V*. Furthermore, *m* is the mass of the adsorbents (g). 

The adsorption kinetics were studied at 25 °C under pH = 6.8 and stirring of 150 rpm. Approximately, 100 mg of the composite adsorbents were dispersed in 50 mL of a 30 mg·L^−1^ Acid Red 66 dye solution. The obtained mixture was stirred at 25 °C and aliquots were withdraw and separated by magnetization. Thereafter, the dye concentrations in the supernatant were determined using a UV-Vis spectrophotometer, at regular time intervals.

The working adsorption isotherm models such as Langmuir and Freundlich equations were applied as described in the literature [16]. The kinetic adsorption was evaluated using the pseudo-first, pseudo-second order kinetic model and Weber–Morris intraparticle diffusion kinetic models, as described elsewhere [8]. 

## 3. Results and Discussion

### 3.1. Structure by XRD, Raman and FTIR

**Figure 1:**
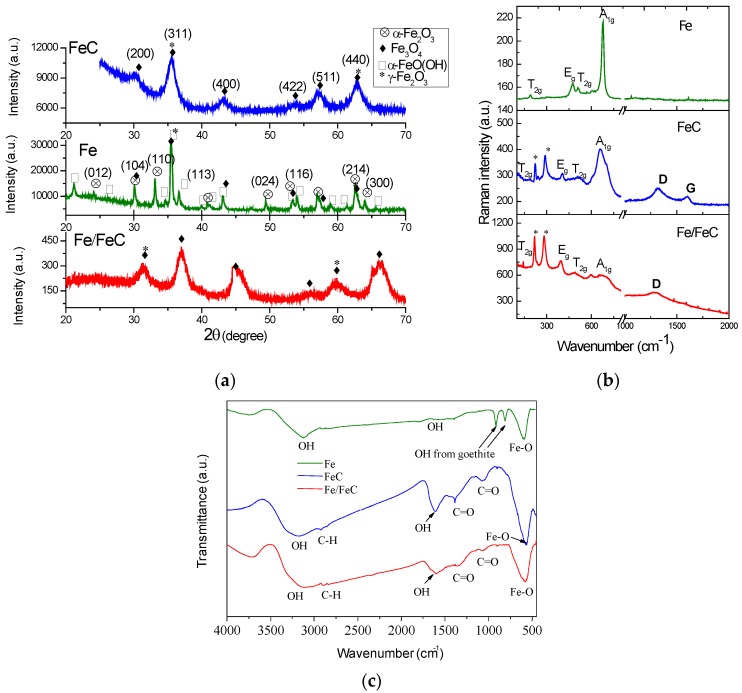
Structural characterizations by (**a**) XRD, (**b**) Raman and (**c**) FTIR measurements. The asterisks mean the presence of the maghemite phase.

XRD diffraction patterns in Figure 1a show the main diffraction crystallographic planes at 2θ = 30.2°, 35.4°, 37.1°, 43.4°, 56.9° and 62.5°, which are attributed to be from the (220), (311), (400), (422), (511) and (440) planes, respectively. These peaks were indexed to a standard pattern of the inverse cubic spinel Fe_3_O_4_ phase, e.g., magnetite with file JCPDS 85-1436, in agreement with some reports [22]. Hence, co-precipitating aqueous solutions of iron sulphate precursors, e.g., Fe^2+^/Fe^3+^ ions mixtures in a basic medium with sucrose promotes the formation of iron hydroxides (Fe(OH)_3_) and oxyhydroxides (α-FeOOH). Subsequently, the former ferrous hydroxides nucleate and grow forming the oxyhydroxides in the presence of OH^−^ ions from ammonia solution and sucrose. Finally, the oxyhydroxides can be converted into magnetite after hydrothermal treatment and drying at 80 °C overnight, as in the case of FeC and Fe/FeC composites. These observations are in accordance with the findings [23,24]. The broad features of the Fe-based composites diffractograms suggest the crystalline nature of the magnetite particles.

Additionally, it is found that the peaks at 2θ = 35.4° (311) and 62.7° (440) are ascribed to the cubic spinel γ-Fe_2_O_3_ phase (maghemite, JCPDS 01-089-5892), which are indeed in similar positions as those of magnetite [25]. As the diffraction peaks are broad due to the quantum confinement effect of the iron particles, the lattice parameters of the magnetite and maghemite phases are closely related and difficult to resolve. Thus, it is not direct to achieve an accurate distinction between magnetite and maghemite phases by powder XRD and hence, a more profound study is beyond motivation and goals of this particular study. Thus, asterisks symbols were used on magnetite patterns to label maghemite as an impurity.

Unlike the composites, which have broad peaks, the pattern of the Fe sample has narrow XRD peaks due to the particles growth and reduction of strain in the lattice originating from defects. Besides, the XRD pattern of Fe sample exhibits poorly crystalline peaks at 2θ values of 21.5°, 33.5°, 36.9°, 41.4°, 53.5° and 59.3° corresponding to the (110), (130), (111), (210), (131) and (211) planes of the hexagonal α-FeO(OH) phase (goethite, JCPDS 03-0249), as found elsewhere [23,26]. Furthermore, peaks at 2θ values of 23.9°, 30.3°, 34.8°, 38.7°, 41.3°, 47.5°, 58.7° and 63.6° unambiguously match well with the (012), (104), (110), (113), (024), (116), (214) and (300) planes of the rhombohedral α-Fe_2_O_3_ phase (hematite) file JCPDS 33-0664. Similar to other observations, the appearance of hematite in the final product is due to hydrolysis of the excessive Fe^3+^ ions, when the Fe^2+^/Fe^3+^ molar ratio present in the solution may be above 2 [24]. Obviously, the diffraction peaks of both magnetite and maghemite facets cannot be ruled out in Fe sample. This result indicates that the preparation method of Fe sample creates a mixture of the hematite, maghemite and magnetite oxides as well as goethite oxyhydroxide with the predominant phase being magnetite with more than 70% of the phase contributions.

Raman spectra of all solids are shown in Figure 1b. Fe sample depicts vibration modes at around at 190 (T_2g_), 304 (E_g_), 532 (T_2g_) and 670 (A_1g_) cm^−1^, all of them attributed to the Fe-O bonds of the Fe_3_O_4_ crystallographic structures [27,28]. Accordingly, the A_1g_ represents the symmetric stretch of oxygen atoms along Fe–O bonds while the Eg and T_2g_ are the symmetric and asymmetric bends of oxygen with respect to the FeO_4_ tetrahedra lattice-vibration, respectively [28]. As aforesaid, magnetite crystallizes in an inverse cubic Fe^3+^(Fe^2+^Fe^3+^)O_4_ spinel structure, which contains Fe^2+^ and Fe^3+^ in the 1:2 ratios and belongs to the *Fd3m* (O_h_^7^) space group. 

Although the co-existence of the hematite, goethite, magnetite and maghemite is observed by the XRD patterns, Raman measurements indicate that the aforementioned vibrational modes confirm the predominance of the Fe_3_O_4_ particles, in agreement with the XRD observations. Regarding the vibrational modes at high frequencies region, no modes are detected indicating the assignment of the sole magnetite structure [28].

In the case of FeC and Fe/FeC samples (Figure 1b), the vibrational modes broaden and blue shift, suggesting the weakness of the Fe-O bonds. This could be assigned to the presence of the quasi-crystalline forms of single carbon layer as it was the case of core-shell arrangement of the nanostructures [29]. The A_1g_ mode of magnetite splits into two weak modes whereas two new modes near 215 and 276 cm^−1^ arise. These modes are indeed totally symmetric, being typical for maghemite as displayed in XRD pattern, because magnetite is isostructural to maghemite with the cubic structure (space group *Fd3m* (O_h_^7^)). As a defective form of magnetite, γ-Fe_2_O_3_ has the Fe atoms occupying two crystallographic distinct sites being tetrahedrally [Fe^2+^] and octahedrally [Fe^3+^] coordinated by oxygen anions from an Fe^3+^(Fe^2+^Fe^3+^)O_4_ structure. 

At high frequencies, it is evident that the broad band at around 1275 cm^−1^ confirms the maghemite presence in Fe/FeC composite probably due to the extra framework γ-Fe_2_O_3_ specie out of the structure. The defect bands from amorphous carbon, namely D band appears superimposed to that of maghemite and thus, the presence of amorphous carbon layer coated on the structure is likely to appear in Fe/FeC sample. 

On the contrary, Raman spectrum of FeC composite depicts the D and G modes at 1342 and 1610 cm^−1^, respectively. The appearance of the D and G bands in the FeC composite suggests that an increased number of structural defects and disorders in the prepared composite in comparison with Fe sample. Noteworthy, XRD patterns of the FeC and Fe/FeC composites do not show distinct diffraction peak of carbon, which implies in a low degree of crystallinity of carbon content of the sample. These observations are confirmed by electron microscopy measurements.

Figure 1c exhibits the FT-IR spectra of the prepared solids. The stretching and bending of the O-H groups from physisorbed water are shown at 3320 and 1615 cm^−1^, respectively. It has been reported that Fe_3_O_4_ particles has crystallized O-H hydroxyl bonded to the unsaturated surface Fe atoms metals in the same positions mentioned above [30]. Besides, the broad bands appearing in all samples at around 550 cm^−1^ are assigned to the Fe–O bonds vibrations in tetrahedral and octahedral sites of magnetite. The above mentioned bands are observable in all solids studied. Importantly, the vibrations at 903 and 800 cm^−1^ in Fe sample are attributed to stretching and bendings of O–H groups, respectively from goethite [31]. It confirms the presence of a mixture of iron oxides, as seen by XRD in Fe sample.

In addition, the absorption bands of the C-H groups stretching are observable at around 2918 cm^−1^ while the symmetrical and asymmetrical stretching vibrations at around 1522, 1337 and 1065 cm^−1^ are attributed to the characteristic bands of carbonyl compounds [32]. All of these bands arise from the decomposition of the sucrose precursors that formed the carbon species evident in the Fe-composites by Raman spectroscopy. This also suggests a strong interaction between the carbon species and Fe_3_O_4_ particles. Therefore, the FTIR and Raman spectra suggest the presence of predominantly magnetite with a small amount of maghemite, as impurity in the composites. For the pure Fe sample, magnetite is 70% and the rest being unambiguously attributed to goethite, hematite, and maghemite. 

### 3.2. Textural Properties and Zeta Potential Determination 

**Figure 2:**
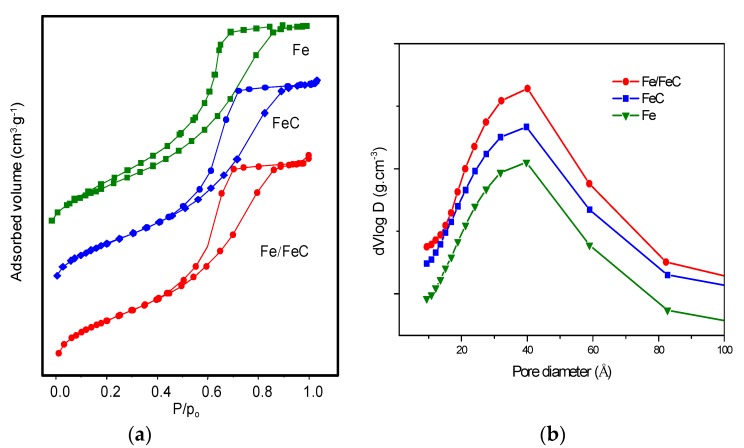
(**a**) Nitrogen adsorption–desorption isotherms and (**b**) pore size distribution curves of the solids studied.

The porosity of the solids is investigated through the N_2_ adsorption–desorption and their BJH pore size distribution analyses. The samples exhibit a typical type-IV isotherm with H_4_ hysteresis loop in the relative pressure range of 0.4–1.0 (Figure 2a). This indicates the presence of characteristic mesoporous materials, as also confirmed in the literature [33].

The textural properties of the solids are obtained from the N_2_ adsorption–desorption and their corresponding BJH pore size distribution analyses (Table 1). Accordingly, the BET surface area of Fe sample is 163 m^2^·g^−1^ with a pore volume of ca. 0.23 cm^3^·g^−1^. 

The elevated textural properties of the solid are attributed to the abundance of void spaces between the interconnected particles during the hydrothermal treatment procedure.

Meanwhile, the textural properties behavior of FeC composite (122 m^2^·g^-1^) suggests that the effect of coexisting carbon species i.g. graphitic and amorphous carbon on the Fe_3_O_4_ cause a decrease of both surface areas and pore volume comparing with those of Fe sample. Simultaneously, the surface area and pore volume of Fe/FeC are greater (202 m^2^·g^−1^ and 0.28 cm^3^·g^−1^, respectively) than those of Fe and FeC samples. This can illustrate the creation of an additional porosity to the FeC solid due to the successful incorporation of iron in the structure, as found elsewhere [34]. That is to say, iron is included in the structure rather than deposited on solid surface. 

Furthermore, the micropores areas, expressed herewith as t-plot areas follows similar trends as those of the mesoporous parameters. On the basis of this result, the solid developed micropores because of gases evolution during the heating processes. It can be concluded samples mainly contains mesoporous and microporous within its matrix.

The pore radius is sharply distributed in a narrow range located at around 27 Å (Figure 2b) revealing that the pore structure is not affected by the considerations mentioned above.

The electrostatic potentials on the surface are shown in Table 2 through the zeta potential values. 

For Fe sample, the negative surface charge of −5.56 mV is developed. On the contrary, the Fe-based composites depict a positively charged surface of +10.8 and +24.9 mV, respectively for FeC and Fe/FeC samples. 

### 3.3. Morphological Aspects of the Solids by SEM-EDS 

**Figure 3:**
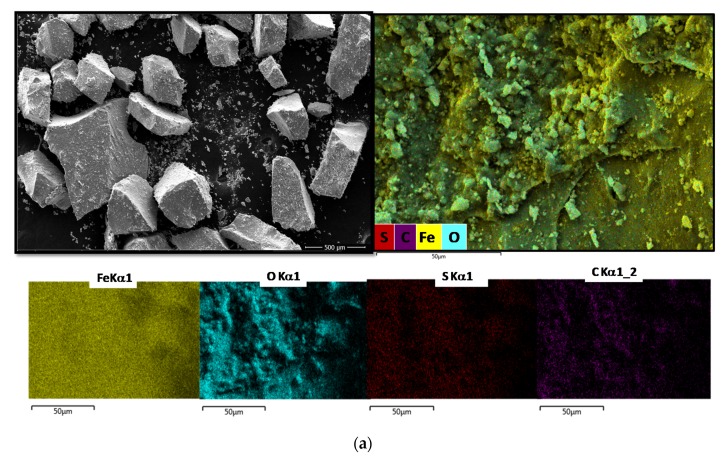

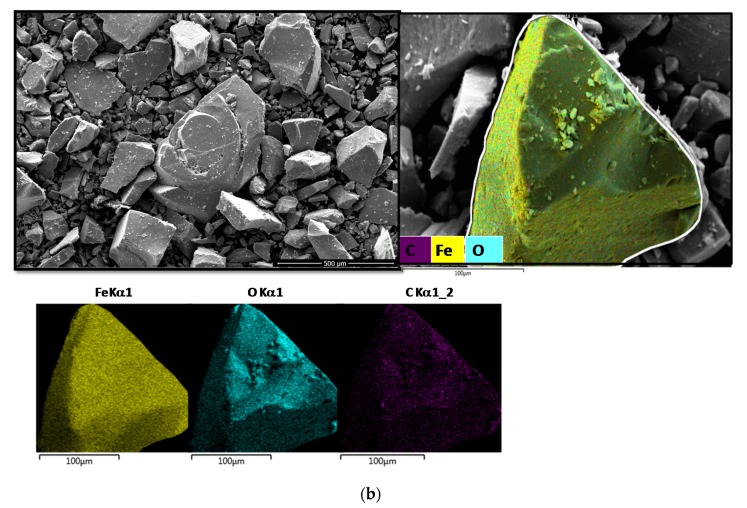
SEM-EDS images of the (**a**) Fe and (**b**) FeC samples.

SEM-EDS analyses are performed to examine the morphological and compositional aspects of the solids (Figure 3).

SEM image of Fe sample shows large particles with non-distinguished shaped and soft surface morphologies (Figure 3a). The magnified image of an individual particle (Figure 3a) depicts numerous aggregates of small particles. 

Moreover, the elemental analysis by surface EDS indicates the composition of Fe and O at 73.1 and 23.9% respectively (Table 2). The impurities from the preparation of the solids such as W and S elements are also observed in a minor amount.

In addition, SEM images for FeC exhibits similar morphologies as those of Fe sample, however in this case all particles tended to agglomerate, as shown in Figure 3b. From EDS as shown in left of Figure 3b, a composition of Fe, O and C being 63.9%, 29.1% and 6.7%, respectively is found, as presented in Table 2. Both Fe species and carbon are present on the surface of the composite, which is consistent with FTIR, XRD and Raman measurements.

**Figure 4:**
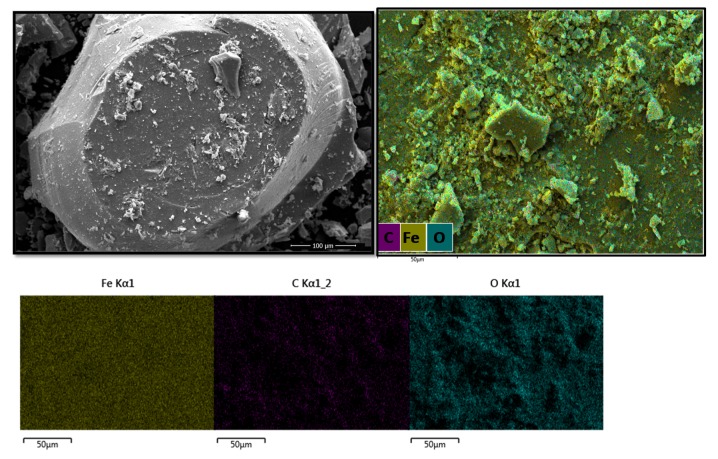
SEM-EDS images of the Fe/FeC solid.

For Fe/FeC sample, the particles possess irregular morphology shapes as depicted in Figure 4 Additionally, the agglomerated composite is quite larger in comparison with other two samples (Figure 4, left side of the panel). 

From EDS, the surface of the solids consists of Fe and C elements indicating that iron is successfully incorporated to the structure, in which Fe^3+^/Fe^2+^ ions form Fe_3_O_4_ and γ-Fe_2_O_3_, besides amorphous carbon as determined by Raman measurements.

### 3.4. Mössbauer, EPR and Magnetization Curves 

**Figure 5:**
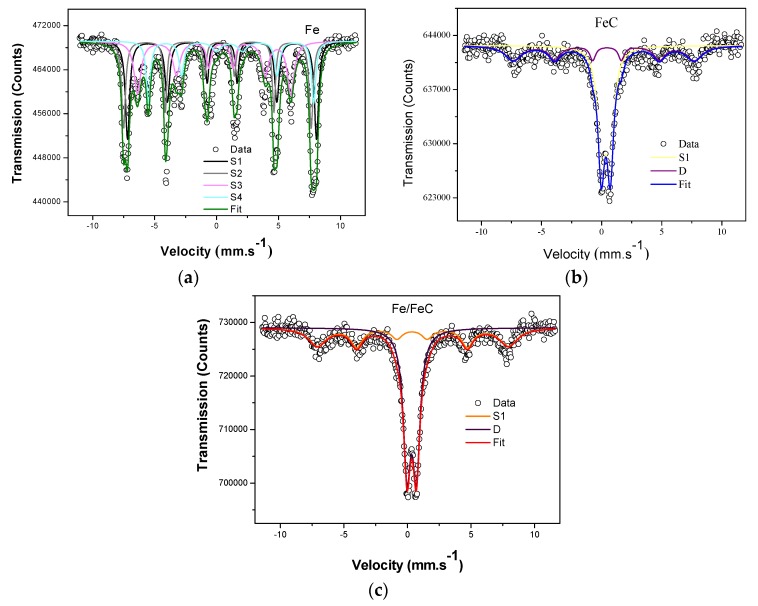
Fitted room temperature Mössbauer spectra of the samples studied (**a**) Fe, (**b**) Fe/C and (**c**) Fe/FeC. The letters S and D represent the sextet and doublets of the curves.

The Mössbauer spectroscopy studies are performed to obtain the chemical environments of the iron on the samples [22,35,36]. Mössbauer spectrum of Fe depicts the typical shape of Fe_3_O_4_ with four sextets, e.g., S1, S2, S3 and S4 (Figure 5a). The S1 corresponds to the hyperfine magnetic field of 47.42 T and δ = 0.44 mm·s^−1^ while S2 has B_HF_ of 47.08 T and δ = 0.15 mm·s^−1^ (Table 3). These kinds of sextets are typical of Fe_3_O_4_ cubic spinel structure with [Fe^3+^]^tetra^ [Fe^2+^Fe^3+^]^oct^O_4_, in agreement with findings on XRD and Raman. The corresponding average parameter for S1 and S2 accounts to 54.7% of the total amount of Fe_3_O_4_ relative abundance. Indeed, S1 sextet suggests that the Fe^3+^ occupies tetrahedral sites whereas the S2 one corresponds to the Fe^2+^/Fe^3+^ in tetrahedral and octahedral sites in non-stoichiometric magnetite [36,37,38]. 

The possibility of maghemite presence cannot be ruled out, since the ^57^Fe hyperfine parameters, isomer shift and quadrupole splitting calculated from the two doublets are close to those of Fe_3_O_4_. This confirms the XRD results. Also, it should be underlined that the Mössbauer parameters in Fe sample are a rather difficult to exclude the possibility of α-Fe_2_O_3_. The two other sextets namely S3 and S4 have hyperfine field of 38.46 and 41.49 T matching well with goethite (α-FeOOH) phase [38]. Accordingly, the parameters of Δ = −0.54 and 0.07 mm·s^−1^ for S3 are assigned to Fe^3+^ ions of goethite, which represents 26.4% of the relative abundance (Table 3).

The FeC spectrum is deconvoluted into a doublet and one sextet, as presented in Figure 5b. The doublet with the parameters such as Δ = 0.74 mm·s^−1^, δ = 0.34 mm·s^−1^ and R.A of 62.3% are typical of superparamagnetic particles of magnetite particles, in accordance to other reports as found in the literature [39]. The remaining relative abundance of ca 37.4% is associated with the sextet possessing the resolved hyperfine parameters of B_HF_ = 46.74 T. Δ = −0.27 mm·s^−1^ and δ = 0.31 mm·s^−1^. 

Thus, carbon content does not change magnetite phase. Only, oxidation states of Fe^2+^ to Fe^3+^ are likely ascribing in maghemite phase for FeC composite.

Similarity, the appearance of one fitted doublet and sextet in the Fe/FeC spectrum (Figure 5c) determines the magnetic structure is composed of both Fe^2+^ and Fe^3+^ from magnetite and maghemite. 

The hyperfine parameter of the doublet is the same as that of the FeC with relative abundance, e.g., RA of 60.2% and the remaining 39.8% for the sextet. This implies that the super-paramagnetic spin relaxation of the particles may take place. Thus, the introduction of additional iron phases in FeC may lead to formation of maghemite coating.

**Figure 6:**
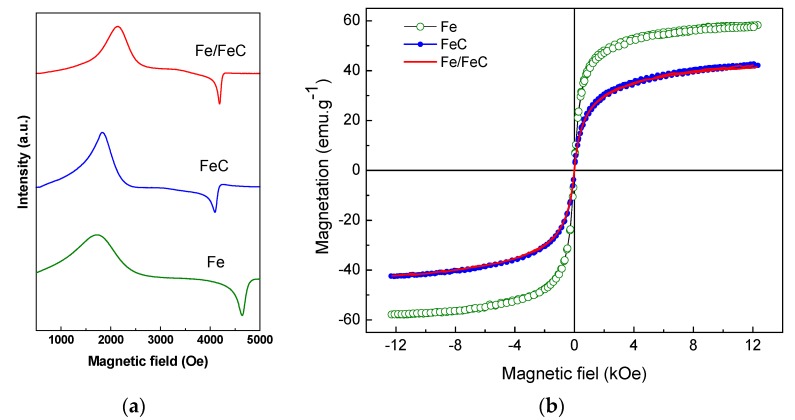
(**a**) EPR spectra and (**b**) Magnetic saturation hysteresis loops of the samples studied.

EPR spectroscopic studies of the solids are performed to directly identify all iron phases. The two broad signals of the X-band electron magnetic resonance spectra are clearly visible for all of samples (Figure 6a). 

The line shapes on EPR spectrum for Fe sample are broader and more asymmetric in comparison with other samples indicating a ferromagnetic resonance. Additionally, a main signal at around 1500 Oe has g-factor of 2.2, which suggest similar distributions of Fe^3+^ species in the solid. These features are frequently observed in other Fe-based samples mainly in hematite phase, as described elsewhere [40,41]. Indeed, the features of the spectra are also typical of cubic magnetocrystalline anisotropy in solids such as magnetite. In accordance with the literature reports, the spectra display the typical cubic magnetocrystalline anisotropy for magnetite-based solids [42,43]. 

The lowest signal at around 4700 Oe depicts g = 4.0 suggesting the paramagnetic resonance of isolated Fe^3+^ with rhombic symmetry, in which the ions are located in tetrahedral FeO_4_ and/or octahedral FeO_6_ local and Fe^2+^ species [40,41,42]. It is important to note that the EPR spectra line shapes of the Fe/FeC and FeC composites are strikingly similar with narrow signals, compared to that of Fe sample. Moreover, the first signal experiences a shift to high values of magnetic fields regions whereas the second one shifts to low regions. This means that the asymmetry ratio values are distinct for all samples. Also, it could be indicative of the negative first order cubic magnetocrystalline anisotropy, in agreement with the literate [43]. This is more evident for Fe/FeC than in FeC that possesses the lowest magnetocrystalline anisotropy field.

Although the EPR spectra of magnetite are still controversial due to the strong absorption of microwave in region, the possible certainties in the assignments are confirmed by the Mössbauer spectroscopy. 

To further understand the nature of the Fe species, the magnetic properties of the solids are obtained by the Vibrating sample magnetometer (VSM). Data of magnetic hysteresis loops (MS) are taken at room temperature, as depicted in Figure 6b. Fe sample has at approximately 58 emu/g, which is much lower than that of the reported value for pristine Fe_3_O_4_, e.g., 92 emu/g [44]. Considering that the existence of non-collinear spins at the surface of Fe_3_O_4_ particles is likely to occur, this might justify the observed low hysteresis value, as previously reported in the literature [36]. The later observations can be associated with the maghemite, hematite and goethite as minority phases in Fe sample. These assumptions are in agreement with coercive field (*H*_c_) value for magnetic particles [34,36]. 

The hysteresis loops and coercive field of the Fe-based composites are reduced by approximately 30%. On the basis of XRD, Raman and Mössbauer data, the magnetic properties can attribute to carbon coating on the magnetic particles. The findings also state that either structural/magnetic transition or anomalous temperature dependence of the magnetocrystalline anisotropy as well as the decreased size of the magnetic structure may cause these behaviors [36]. 

### 3.5. Adsorption Studies

#### 3.5.1. Equilibrium Study

**Figure 7:**
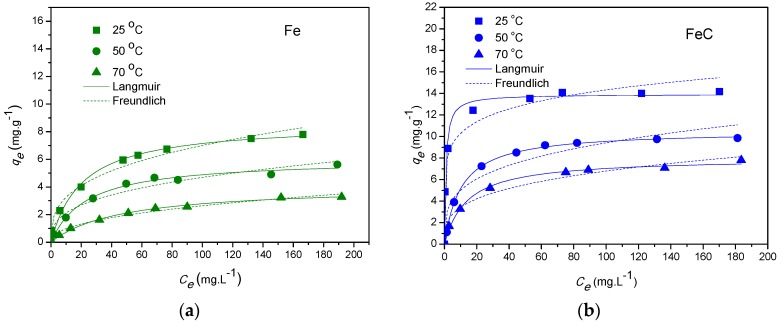

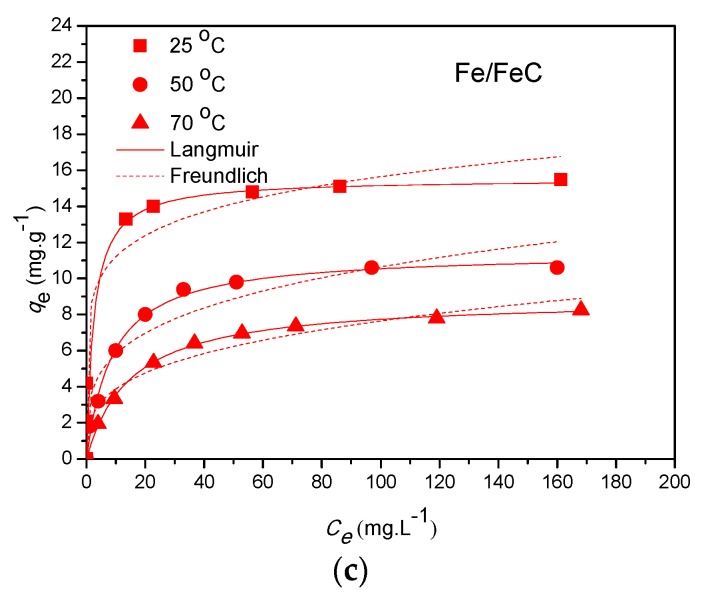
Langmuir and Freundlich plots at different temperatures for Acid Red 66 adsorption on (**a**) Fe, (**b**) Fe/C and (**c**) Fe/FeC.

Typical Langmuir and Freundlich models for adsorption isotherms are used to illustrate the equilibrium between the dye solution and the adsorbents, as shown in Figure 7.

For the Langmuir isotherm model, the adsorption occurs at a specific uniform location within the adsorbent and a monolayer is formed on the solid surface, as indicated in the literature [12,45]. 

The equilibrium considerations on the isotherm equation are expressed as:

(2) $$q_{e}=\frac{q_{o}\cdot b\cdot C_{e}}{\left(1+bC_{e}\right)}$$

where *q_e_* (mg·g^−1^) and *C_e_* (mg·L^−1^) are the equilibrium adsorption capacity of the adsorbate and the equilibrium concentrations of the adsorbent, respectively. *q_o_* is the maximum amount of the adsorbent per unit mass of sorbent to form a complete monolayer on the surface bound at high *C_e_. b* is the Langmuir adsorption equilibrium constant, which is related to the affinity of the binding sites (L·mg^−1^) [46,47]. 

The Langmuir isotherms show that the temperature increment is not favorable to the adsorption process (Figure 7), as an intrinsic characteristic of the exothermic adsorption process. 

Thus, the dye adsorption at ambient temperature (25 °C) conditions is more favorable to occur. Accordingly, the values of the adsorption of Acid Red 66 are 8.61, 13.92 and 15.53 mg·g^-1^ for Fe, FeC and Fe/FeC samples, respectively (Table 4). Moreover, FeC and Fe/FeC have best results for *b* values at room temperature, which further demonstrates an excellent selective adsorption capacities of the two composites.

The empirical equation describes heterogeneous systems for Freundlich model isotherm analysis, being expressed as:

*q_e_* = *K_F_*·C_e_^1/*n*^ (3)

where *q_e_* is the maximum adsorption capacity amount (mg g^-1^); *C_e_* is the equilibrium concentration of the adsorbate (mg·L^−1^). *K_F_* is the Freundlich adsorption equilibrium constant. *n* is the constant adsorption strength, where a favorable range of 1/*n* is between 0.01 and 0.435, well described in references [46,47,48].

Both the Langmuir and Freundlich adsorption equilibrium models use a non-linear regression model, according to the Levenberg–Marquardt algorithm [49]. In addition, the standard deviation values (*SD*) are obtained as follows:

(4) $$SD=\sqrt{\frac{\sum {\left[\left(q_{t,exp}-q_{t,cal}\right)/q_{t,exp}\right]}^{2}}{\left(N\;-\;1\right)}}$$

where *N* is the number of the equilibrium data; *q_t,exp_* and *q_t,cal_* represent the experimental and calculated equilibrium points. 

It is possible to determine a non-temperature dependence for the adsorption, since the fitting curves with Freundlich isotherms follow similar behavior with an optimal at 25 °C, as displayed in Figure 7. The 1/*n* values are comparable lower values than unity for all solids, which indicates that the Freundlich model agreed reasonably well at low adsorbate concentrations. However, both of aforesaid models do not adequately explain the adsorption of the dye Acid Red 66 on the adsorbents at high adsorbate concentrations due to the surface coverage, as found elsewhere [50].

In addition, the results in Table 4 illustrates that the Langmuir adsorption isotherm fits better to all experimental data, as shown by the values of correlation coefficient (0.919 < R^2^ < 0.999). This means that the *SD* parameters of the Langmuir adsorption model keep low values having *SD* values between 0.009 and 0.065 under the same experimental conditions. The theoretical and experimental data agree well when comparing with *SD* value in between 0.011 and 0.091. 

#### 3.5.2. Thermodynamic Studies

The thermodynamic parameters such as adsorption enthalpy (Δ*H*), entropy (Δ*S*) and Gibbs Energy (Δ*G*) are estimated to provide the information of the bond strengths between the adsorbent surfaces and Acid Red 66 molecule. 

The estimation of Δ*H*, Δ*S* and Δ*G* in single-component system is achieved by the following equations:

(5) $$C_{s}=C_{0}-C_{w}$$

(6) $$K_{ad}=\left(C_{0}-C_{w}\right)/C_{w}$$

(7) $$\Delta G^{o}=-RT\left(lnK_{ad}\right)$$

(8) $$ln(K_{ad})=-\frac{\Delta H^{o}}{RT}+\frac{\Delta S^{o}}{R}$$

where *K_ad_* is the adsorption coefficient represented by the equilibrium concentration of the adsorbate on the solid (*C_s_*, mg·L^−1^), C_o_ is the initial concentration and the equilibrium concentration of the adsorbate in solution (*C_w_*, mg·L^−1^), *R* is the gas universal constant (8.314 J·mol^−1^·K^−1^) and *T* is the absolute temperature (K). 

**Figure 8:**
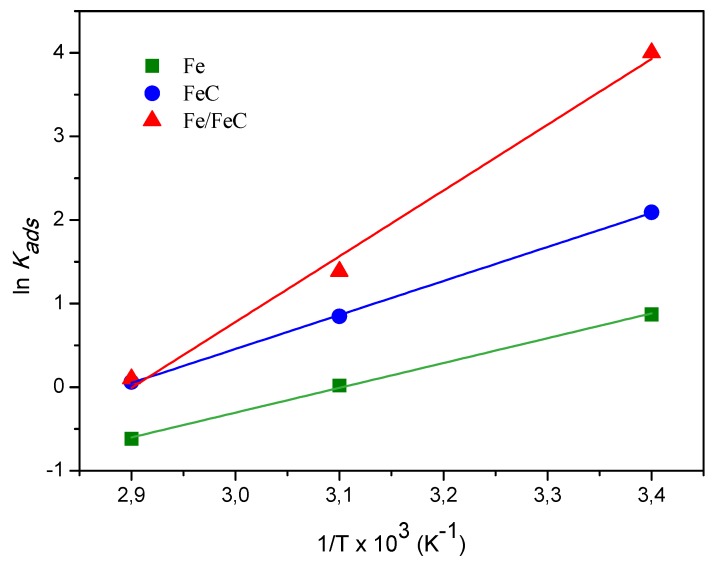
Vant’ Hoff plots for Acid Red 66 adsorption on (**a**) Fe, (**b**) FeC and (**c**) Fe/FeC.

The *ΔG* values at distinct temperatures of 25 °C, 50 Thermo C e 70 °C are obtained from *K_ad_* parameter from Equation (6) while *ΔH* e *ΔS* values are predicted from the ln *K_ad_* versus 1/*T* plot (Figure 8). The ln *K_ad_* versus 1/*T* plots for Langmuir and Freundlich isotherms follows a linearity. The thermodynamic parameters are summarized in Table 5.

The calculated adsorption heat obtained are −28.1, −38.5, and −74.3 kJ·mol^−1^ for the Acid Red 66 adsorption of Fe, FeC and Fe/FeC, respectively. These negative values confirm the feasibility and exothermicity of adsorption. This also illustrates a high preference for Acid Red 66 dye to adsorb chemisorption on the FeC and Fe/FeC composites.

The negative values for Δ*G* confirm the spontaneity of the adsorption of Acid Red 66 over FeC and Fe/FeC composites, in good agreement with the literature reports that shows the physisorption occurrence over ΔG values in the range of 0 to −20 kJ·mol^−1^ [51,52]. 

This also illustrates a preference for Acid Red 66 dye to adsorb by either chemisorption or physisorption processes on the composites. In agreement, the findings states that the mean free energy of sorption in the E < 8.0 kJ·mol^−1^ range represents the physisorption process while 8.0 ≤ E ≤ 16.0 kJ·mol^−1^ indicates the ionic exchange and the E > 16.0 kJ·mol^−1^ suggests the chemisorption occurrence during the adsorption of Acid Orange 7 (AO7) over chitosan-based magnetic adsorbents [52]. Interestingly, Δ*G* values decrease with the increase of adsorption temperature with Fe sample at 70 °C being an exception. Comparable results are obtained for the adsorption of Acid Red 66 onto carbon nanotubes [53].

Another observation is the fact that the solution is at the isoelectric point with pH = 6.8 and thus, the hydroxyl groups and sulfonic groups from the dye are weakly interacting with the surface of the Fe sample causing repulsions, in some extension (ζ = −5.56 mV, Table 2). In contrast, the surface of the Fe-based composites has positive charges (ζ = +10.8 mV for FeC and ζ = +24.9 mV for Fe/FeC at the same pH value. This may promote the interactions among the negative charge of the –SO_3_^−^ and –N=N– groups of the azo dye and the composites. Therefore, the adsorption capacity follows the order Fe/FeC > FeC > Fe, according to the arranged chemical system for adsorption of Acid Red 66. 

#### 3.5.3. Adsorption Kinetics

**Figure 9:**
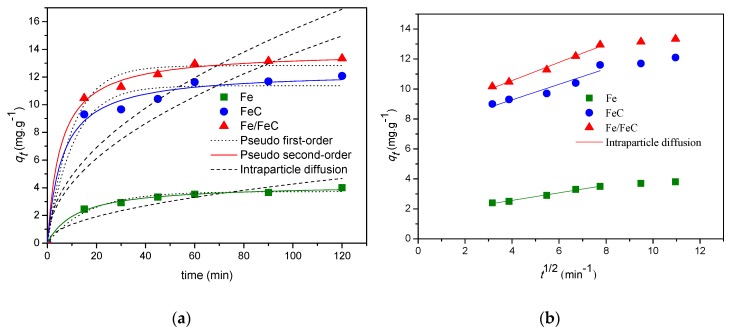
Kinetics analyses of the adsorption of Acid Red 66 on the solids studied: (**a**) Non-linear plots for pseudo1st order kinetics, pseudo 2nd order kinetics and intraparticle diffusion model. (**b**) Linear plot model for intraparticle diffusion. The initial concentration of the dye is 30 mg·L^−1^, pH = 6.8, T = 25 °C and dosage of the adsorbent of 2 g·L^−1^.

Plot of the equilibrium of the dye adsorption versus the contact time (Figure 9) for distinct concentration of the Acid Red 66 dye is shown. The Figure 9a depicts that dye adsorption increase remarkably during first initial minutes until a certain concentration point and then, slowly rises over the course of 120 min. This is an indicative of the dye molecules are absorbed during the first minutes of reaction; a further occupation of the adsorption sites of the solids is seen with the consequent decrease of the interactions between the adsorbent and azo dye, as the reaction proceeds.

Considering the influence of the solids on the dye adsorption, the Fe sample exhibits the lowest discoloration capacity, among the solids studied. Accordingly, the extent of Acid Red 66 removal over the time demonstrates that the Fe sample has 52.6% of the removal efficiency, while FeC gives nearly two times more. Also, the Fe/FeC holds 98.4% from the equilibrium percent removal, as clearly demonstrated in Table 6.

**Figure 10:**
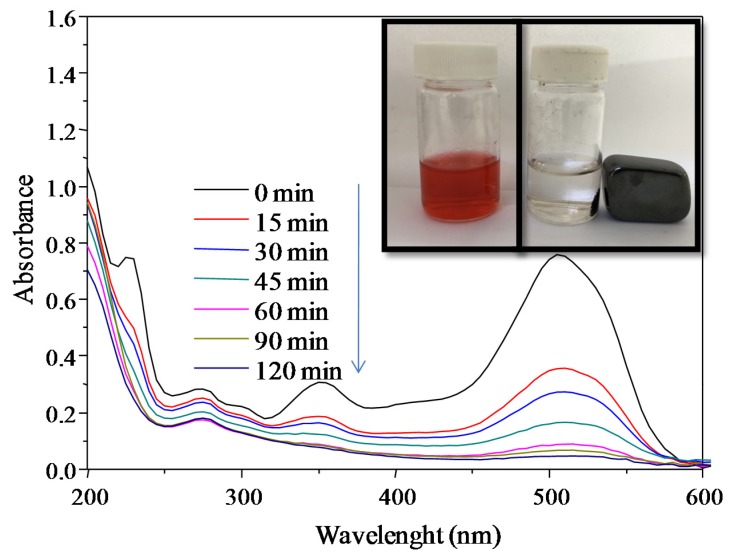
UV-Vis absorption spectra for Acid Red 66 at different time intervals under visible light irradiation for FeC composite. The included Figure is the picture of the dye solution before (0 min) and after (120 min) adsorption processes.

To illustrate the abovementioned results, the UV-Vis profiles of Fe/FeC sample depict absorbance spectrum after the adsorption at different reaction times (Figure 10). The absorbance bands at 510, 350 and 278 nm are observed for the red glow tone of the Acid Red 66 dye solution (Figure 10, inset). These bands are completely vanished from the spectra at 120 min of dye decolorization. Indeed, the solution became clear and the adsorbent is subsequently removed from the solution by using a conventional ferromagnet bar.

This indicates that decolorization of Acid Red 66 occurs quickly because of the deconstruction of the conjugated aromatic structures of the dye.

One key factor governing the efficiency of adsorption could be attributed to structural properties of the solid sample, although the literature reports indicate that the adsorption of anionic dyes onto α-Fe_3_O_3_, γ-Fe_3_O_3_ and α-FeO(OH) occurs as isolated phases [49,54]. However, the results obtained demonstrate that the mixture of the abovementioned phases in Fe sample does not display good adsorption capacities.

Judging from the fact that about 70% of Fe_3_O_4_ is present in Fe sample (XRD, Mössbauer and EDS results), the magnetite phase is likely prevalent for the adsorption phenomena.

Apparently, the anionic azo dye adsorption takes place primarily in the OH groups responsible for the negative charge of Fe from the accessible cubic Fe_3_O_4_ structure through the sulfate groups of the dye. This is in accordance with the findings from Mössbauer and XRD techniques and in line with the findings [16]. The saturation of the adsorption capacity of Fe_3_O_4_ particles may occur and thus, the dye adsorption capacity onto Fe sample is low, when the time of reaction passed.

The composites might be able to capture azo dye due to its diffusion through carbon matrix porosity; this occurs due to the following factors: (i) electrostatic interactions between –SO_3_^–^ group as contained on the dye and Fe^3+^/Fe^2+^ species phase over Fe/FeC and FeC composites; (ii) the π-π interaction between the azo dye aromatic structure (or even –N=N– groups) and OH groups as shown in Fe_3_O_4_, since this hydroxyl groups appear on FTIR results spectroscopic signals and (iii) a possible electrostatic attraction between the azo dye structure and positive charges over composites surface; the later being confirmed by the positive charges found by zeta potential techniques.

At this point, the structural, textural and morphological properties of the Fe composites undermine their high adsorption capability towards the Acid Red 66 dye.

In summary, the adsorption data reflects better capacity of Fe-carbon composites, when comparing with Fe sample.

##### Pseudo-First and Second Order Models

The efficiency of adsorption can be described in terms of kinetic models via Lagergren pseudo-first-order and pseudo-second-order equations. The first-order kinetic model is extensively applied for the adsorption of dyes by using Equation (8), as described in the literature [46,54]:

(9) $$q_{t}=q_{e}\left(1-e^{-k_{1}t}\right)$$

where, *q_e_* and *q_t_* are the adsorption capacity at the equilibrium and the individual capacity at various time, respectively. *k*_1_ (min^−1^) is the rate constant of the pseudo-first-order model of adsorption process and *t* is the time in minutes.

In the case of the pseudo-second-order model, the chemisorption is the rate-limiting step while adsorption may take place on the sites where no interactions among the adsorbates occur. The equation is expressed as:

(10) $$q_{t}=\frac{t}{\frac{1}{k_{2}q_{e}^{2}}+\frac{t}{q_{e}}}$$

where *k*_2_ (g·mg^−1^·min^−1^) is the rate constant of pseudo-second-order adsorption.

According to Figure 9b, the initiation of decolorization in the solution occurs faster during the first 10 min. with 60% of the dye being removed from the solution, when Fe sample was tested; the decolorization is close to 80% for Fe-carbon based composites. However, a maximum adsorption capacity is reached with 95% of the dye removal at 20 min of reaction.

For easy understanding the kinetic parameters of the pseudo-first-order and pseudo-second-order models, the data are shown in Table 6. The parameters calculated from the pseudo-first-order model have R^2^ < 0.968 and *SD* in the range of 0.045–0.067. This suggests that the pseudo-first-order model is less accurate, which is in agreement with previous studies of pseudo1^st^ order model as applied to dye adsorption process data [49].

Conversely, R^2^ values of second-order kinetic model are above 0.987 with the *SD* in the range of 0.022–0.043. Thus, best results are achieved by using pseudo-second-order model, as found for anionic dyes [45,55,56,57,58]. Furthermore, the rate constant of adsorption (*k*_2_) is superior to 0.0133 g·mg^−1^·min^−1^ with Fe/FeC holding the highest *k*_2_ values due to the rapid dye adsorption and removal.

##### Intra-Particle Diffusion

The mechanisms and rate controlling steps the kinetics of adsorption is investigated through the intraparticle diffusion model from data (Figure 9b). The model proposes that a surface diffusion mechanism governs adsorption, in a first stage while the pore diffusion mechanism may occur in second stage, according to the following Equation (10) [45,56]:

(11) $$q_{t}=k_{int}\sqrt{t}+C$$

where *k_int_* is the intra-particle diffusion rate constant (mg·g^−1^·min^−1/2^), which can be analyzed from the slope of the linear plot of *q_t_* versus *t*^1/2^ and *C* is the intercept of the graph, which gives the thickness of the boundary layer.

The curves do not cross zero value (Figure 9b) indicating that some other adsorption mechanism like surface diffusion in combination with intraparticle diffusion is occurring at rate controlling step, as shown in Table 7.

To corroborate these results, the deviation at the origin can be attributed to a variation of mass transfer at initial and final stages during Acid Red 66 adsorption process [56].

### 3.6. XPS Measurements of the Spent Adsorbents

The examination of the chemical states of Fe and C elements in the samples by XPS provides additional information about the chemical composition at the surface and the chemical state of the elements, after the adsorption process. Table 8 shows the binding energy (in eV) of the selected photoemissions and in the case of the C *1s* and O *1s* signals, the % of each area of the different contributions of the deconvoluted spectra are also included (in brackets).

The survey XPS spectrum of the Acid Red 66 dye shows the presence of the C, O, S, Na, Cl and N elements. The C 1*s* core level spectrum can be deconvoluted into four components corresponding to –C=C– and adventitious carbon (284.8 eV (70%)), C–OH (285.7 eV (9%)), C–N=N (286.7 eV (17%)) and C–SO_3_^−^ (288.2 eV(4%)) [59,60]. The S 2*p* core level spectrum shows a doublet S 2*p*_3/2_ and S 2*p*_1/2_ at 168.1 and 169.3 eV owing to the sulfate groups of the dye. Interestingly, the corresponding peak position on the residual sodium appears with BE at 1071.8 eV pointing to charge compensating the sulphate group. Obviously, the nitrogen to nitrogen bond of the dye is observed at 399.9 eV. Furthermore, The O 1*s* core level spectrum shows two contributions at 531.8 eV (47%) and 533.1 eV (53%) assigned to –SO_3_^−^ and C-OH groups, respectively [59].

According to the data from XPS, the solids after dye adsorption contain Fe, C, O and N elements. In the case of Fe sample, the binding energies (BE) located at about 710.5–711.0 and 724.2 eV are ascribed to Fe 2*p*_3/2_ and Fe 2*p*_1/2_, respectively.

This indicates that Fe species exist as Fe^3+^ and Fe^3+^ coming from the γ-Fe_2_O_3_ and Fe_3_O_4_ besides that of α-Fe_2_O_3_ phases [22]. Moreover, the shake satellite peak at 718.8 eV is associated with the γ-Fe_2_O_3_. The O *1s* core level spectra exhibit contributions at 530.0 and 531.5 eV. While the first contribution is assigned to lattice oxygen from iron oxides, the latter is due to surface hydroxyl groups and oxygen bonded to carbon [22]. These results confirm that the samples do not suffer from phase transformation and the α-Fe_2_O_3_, γ-Fe_2_O_3_ and Fe_3_O_4_ phases are preserved, after the adsorption process.

The C 1*s* core level spectrum depicts three contributions with BE values at 284.9 eV (82%), 286.4 eV (8%) and 288.6 eV (10%) being assigned to C=C and adventitious carbon, C–O and sulfonate species, from the adsorbed dye on solid surface. Accordingly, the atomic concentration of the elements for spent Fe sample is C: 23.3%, N: 0.33%, O: 51.75%, Fe: 24.3% and 0.30% of sulfur. Judging from the fact that the dye possesses all these chemical entities, a strong adsorption of the dye on solid surface is likely. This is in accordance with adsorption studies results that suggest the chemisorption of the dye onto the sample Also, a limited amount of carbon species on surface is found, as a consequence of the saturation of the Fe adsorbent with the dye.

Specifically, both the binding energies of Fe 2*p*_3/2_ and Fe 2*p*_1/2_ relative to that of the FeC sample after dye adsorption show similarities with that of Fe sample. Also, the BE are due to the Fe^3+^ and Fe^2+^ species present on the surface. Reasons for this phenomenon can be clearly related to the fact of the stability of the Fe_3_O_4_ (γ-Fe_2_O_3_) phase in the spent FeC composite. Accordingly, the BE at 529.9, 531.4 and 532.8 eV from O *1s* can be ascribed to the lattice oxygen of Fe species and oxygen surface hydroxyl groups and from the adsorbed dye. Moreover, the C 1*s* and N 1*s* binding energies are similar to those of their Fe oxide counterpart, implying that the FeC and the dye interaction are relatively strong. The surface atomic concentration is C: 52.33%, N: 10.94%, O: 30.33%, Fe: 6.05% and S: 0.35%. Such composition indicates that the amount of carbon and nitrogen are much higher than that in the Fe sample after dye adsorption due to the fact that the dye adsorption is greater on the FeC composite, as shown in the adsorption studies results. However, the % of N on the FeC surface is higher than that that of Fe sample, which indicates the probably dye adsorption takes on the later sample.

As for the FeC sample, Fe/FeC sample after dye adsorption has similar values of C *1s*, O *1s* and N *1s* binding energies range to FeC. On the other hand, the composition is slightly different, e.g., C: 47.77%, N: 8.26%, O: 35.58%, Fe: 8.218% and traces of ca. 0.17% from sulfur, which shows a smaller amount of the C, N, S and O from the dye on the surface of the adsorbent. This lower surface content of the elements from the dye is due to the higher surface area of this adsorbent (202 m^2^·g^−1^). In this way part of the organic dye is into the mesopores of the solid and cannot be detected by XPS. Thus, the availability of the Fe species could explain the better performance of Fe/FeC towards the adsorption of acid Red 66, compared with Fe and FeC analogues.

## 4. Conclusions

The synthesis of magnetic FeC and Fe/FeC composite adsorbents was performed using hydrothermal methods in the presence of sucrose. The solids were tested for Acid Red 66 dye removal in aqueous solution. The characterization techniques depicted the porous FeC composite structure and Fe_3_O_4_ particles coated by a carbon matrix. In the case of Fe/FeC, the porous FeC structure possesses Fe_3_O_4_ particles coated by a carbon matrix and γ-Fe_2_O_3_ particles. The equilibrium results were best fitted using the Langmuir model with an adsorption capacity of 15.53 mg·g^−1^ at room temperature over Fe/FeC. The kinetic studies indicate that the adsorption process followed the pseudo-second order model, which suggested a mechanism of chemisorption. The fast adsorption rate over Fe/FeC is assigned to possible available active binding sites with Fe^2+^/Fe^3+^ over Fe_3_O_4_ particles’ surface and hydrogen bonding between anionic Acid Red 66 dye and adsorbence.

## Figures and Tables

**Table 1:** Textural properties of the solids obtained from nitrogen adsorption–desorption isotherms.

Sample	BET Surface Areas (m^2^·g^−1^)	Pore Volumes (cm^3^·g^−1^)	t-Plot Surface Areas (m^2^·g^−1^)	Pore Sizes (Å)
Fe	163	0.23	170	27
FeC	122	0.17	126	27
Fe/FeC	202	0.28	205	28

**Table 2:** Zeta potential and composition by EDS measurements of the solids studied.

Sample	Zeta Potential (mV)	Composition (wt%)			
		Fe	C	O	S
Fe	−5.56	73.1	–	23.9	0.1
FeC	10.8	63.9	6.7	29.1	–
Fe/FeC	24.9	62.6	6.7	30.0	–

**Table 3:** Mössbauer parameters obtained from refinements of the spectra at 77 K. Electric quadrupole splitting (Δ), isomer shift (δ), hyperfine magnetic field (B_HF_) and Relative spectral area (R.A) parameters of the Fe-containing samples. The letter S represents the sextets in the samples.

Samples	Electric Quadrupole and Isomer Shift			
	B_HF_ (T)	Δ (mm/s)	δ (mm/s)	R.A. (%)
Fe (S1)	47.42	−0.01	0.44	30.2
Fe (S2)	47.09	−0.18	0.15	24.5
Fe (S3)	38.46	−0.54	0.07	26.4
Fe (S4)	41.60	0.13	1.02	18.8
Fe/FeC (S1)	46.83	0.08	0.44	39.8
Fe/FeC (D)	–	0.73	0.33	60.2
FeC (S1)	46.74	−0.27	0.31	37.4
FeC (D)	–	0.74	0.34	62.6

**Table 4:** Parameters of the Langmuir and Freundlich isotherm models for the adsorption the Acid Red 66 dye over the samples at distinct temperatures.

Adsorbent	Temperature	Langmuir				Freundlich			
	(°C)	*q_o_* (mg·g^−1^)	*b* (L·mg^−1^)	R^2^	*SD*	*K_F_* (mg^1−1/n^·L^1/n^·g^−1^)	*1*/*n*	R^2^	*SD*
Fe	25	8.616	0.049	0.989	0.065	1.512	0.333	0.981	0.086
	50	6.030	0.043	0.991	0.034	0.954	0.345	0.951	0.091
	70	4.080	0.022	0.995	0.054	0.361	0.435	0.981	0.061
FeC	25	13.92	1.125	0.968	0.009	7.394	0.011	0.975	0.011
	50	10.54	0.096	0.999	0.009	2.661	0.270	0.923	0.083
	70	8.007	0.072	0.994	0.022	1.732	0.294	0.973	0.051
Fe/FeC	25	15.53	0.404	0.919	0.011	8.025	0.145	0.972	0.066
	50	11.46	0.114	0.993	0.014	3.174	0.263	0.914	0.091
	70	8.901	0.067	0.999	0.009	1.972	0.294	0.949	0.067

**Table 5:** Thermodynamic parameters the adsorption the Acid Red 66 dye over the samples at distinct temperatures.

Adsorbent	Δ*H^o^* (kJ·mol^−1^)	Δ*S^o^* (J·mol^−1^·K^−1^)	R^2^	Δ*G^o^* (kJ·mol^−1^)		
				25 °C	50 °C	70 °C
Fe	−28.1	−86.9	0.998	−2.16	−0.05	1.77
FeC	−38.5	−111.7	0.999	−5.18	−2.27	−0.17
Fe/FeC	−74.3	−216.9	0.988	−9.91	−3.72	−0.29

**Table 6:** Kinetic parameters at 25 °C for Acid Red 66 adsorption on the solids.

Adsorbent	Dye Removal (%)	*q_e_* (mg·g^−1^)	Pseudo 1st Order				Pseudo 2nd Order			
			*k*_1_ (min^−1^)	*q_e_*_1_ (mg·g^−1^)	R^2^	*SD*	*k*_2_ (g·mg^−1^·min^−1^)	*q_e_*_2_ (mg·g^−1^)	R^2^	*SD*
Fe	52.6	4.001	0.014	3.720	0.976	0.055	0.0094	4.249	0.994	0.024
FeC	73.3	12.13	0.022	11.37	0.968	0.067	0.0132	12.40	0.987	0.043
Fe/FeC	98.4	13. 31	0.024	12.82	0.985	0.045	0.0134	13.85	0.997	0.022

**Table 7:** Intraparticle diffusion kinetic parameter at 25 °C for Acid Red 66 adsorption on the solids.

Adsorbent	Intraparticle Diffusion		
	*k_int_* (mg·g^−1^·min^−1/2^)	R^2^	*SD*
Fe	0.427	0.831	0.183
FeC	1.367	0.687	0.234
Fe/FeC	1.542	0.647	0.244

**Table 8:** XPS data obtained from the spectra of the solids.

Sample	Binding energy (eV)							
	Fe 2*p*_3__/2_	Fe 2*p*_1__/2_	S 2*p*_3/2_	Na 1*s*	Cl 2*p*_3/2_	N 1*s*	C 1*s*	O 1*s*
Fe	710.8	724.2				399.6	284.9(82)	530.0(68)
							286.4 (8)	531.5(32)
							288.6 (4)	
FeC	710.9	724.3	168.1			399.7	285.0(45)	529.9(44)
							286.1(43)	531.4(36)
							288.0(12)	532.8(20)
Fe(FeC)	710.6	724.3	168.1			399.7	285.0(50)	530.4(48)
							286.2(38)	531.5(35)
							288.1(12)	532.9(17)
Acid Red dye			168.1	1071.8	198.2	399.9	284.8(70)	
							285.9 (9)	
							286.7(17)	531.8(47)
							288.3 (4)	533.2(53)
